# New progresses on cell surface protein HSPA5/BiP/GRP78 in cancers and COVID-19

**DOI:** 10.3389/fimmu.2023.1166680

**Published:** 2023-05-18

**Authors:** Ting Li, Jiewen Fu, Jingliang Cheng, Abdo A. Elfiky, Chunli Wei, Junjiang Fu

**Affiliations:** ^1^ Key Laboratory of Epigenetics and Oncology, the Research Center for Preclinical Medicine, Southwest Medical University, Luzhou, Sichuan, China; ^2^ Biophysics Department, Faculty of Science, Cairo University, Giza, Egypt

**Keywords:** HSPA5, expression, cancer, SARS-CoV-2, COVID-19, therapeutics, natural products

## Abstract

Heat-shock-protein family A (Hsp70) member 5 (HSPA5), aliases GRP78 or BiP, is a protein encoded with 654 amino acids by the HSPA5 gene located on human chromosome 9q33.3. When the endoplasmic reticulum (ER) was stressed, HSPA5 translocated to the cell surface, the mitochondria, and the nucleus complexed with other proteins to execute its functions. On the cell surface, HSPA5/BiP/GRP78 can play diverse functional roles in cell viability, proliferation, apoptosis, attachments, and innate and adaptive immunity regulations, which lead to various diseases, including cancers and coronavirus disease 2019 (COVID-19). COVID-19 is caused by the severe acute respiratory syndrome coronavirus 2 (SARS-CoV-2) infection, which caused the pandemic since the first outbreak in late December 2019. HSPA5, highly expressed in the malignant tumors, likely plays a critical role in SARS-CoV-2 invasion/attack in cancer patients *via* tumor tissues. In the current study, we review the newest research progresses on cell surface protein HSPA5 expressions, functions, and mechanisms for cancers and SARS-CoV-2 invasion. The therapeutic and prognostic significances and prospects in cancers and COVID-19 disease by targeting HSPA5 are also discussed. Targeting HSPA5 expression by natural products may imply the significance in clinical for both anti-COVID-19 and anti-cancers in the future.

## 1 Introduction

Heat-shock-protein family A (Hsp70) member 5 (HSPA5) (OMIM: 138120), aliases glucose-regulated protein 78 (GRP78) or binding immunoglobulin protein (BiP), encodes 654 amino acids by the *HSPA5* gene. The *HSPA5* gene (GenBank no.: NM_005347.5) is cytogenetic and located on human chromosome 9q33.3. Like other heat shock proteins, this protein is usually resident in the endoplasmic reticulum (ER), a continuous membrane system within the eukaryotic cell cytoplasm. When the ER was stressed, HSPA5/BiP/GRP78 translocated to the cell surface, the mitochondria, and the nucleus that complexes with other proteins to execute its functions. As a master chaperone protein, HSPA5 responds in the ER when misfolded or unfolded proteins accumulate (1) and involves in the degradation of misfolded proteins or correct folding initially *via* interacting with DnaJ heat shock protein family (Hsp40) member C10 (DNAJC10), another ER-resident chaperone protein, facilitating its release from substrates.

In those stressed cells, HSPA5 is translocated to the cell surface (cs-HSPA5), thus binding to numerous ligands and activating various intracellular signaling/pathways. On the cell surfaces, HSPA5 could play diverse function roles, including cell viability, proliferation, apoptosis, attachments, and regulations of innate and adaptive immunity (2). Dysregulation of HSPA5 is associated with various diseases, such as cancers, cardiovascular diseases, immunological diseases, obesity, neurodegenerative diseases, and stroke. As we known, coronavirus Disease 2019 (COVID-19) is caused by the infection of the severe acute respiratory syndrome coronavirus 2 (SARS-CoV-2) virus, which has aroused the pandemic since the first outbreak in late December 2019. A great number of patients suffer from the disease with defenseless immunity, including people with cancers. Malignant tumors go by the name of the second killer of human disease, bringing a difficult disaster for many families. People who burden the double miseries are an important vulnerable group, arousing the attention of the majority of scientists. Therefore, we are urgent to find new potential targets for tricky diseases. Additionally, it was reported that cs-HSPA5 is responsible for many infectious diseases, including mucormycosis, Japanese Encephalitis, and COVID-19, through different pathways or targets (3–6).

Mucormycosis is a serious but uncommon fungal infection caused by Rhizopus species (mainly R. *oryzae*). In contrast, Japanese encephalitis is a viral infection that targets the brain, while COVID-19 was caused by betacoronavirus called SARS-CoV-2. SARS-CoV-2 is a human coronavirus like the previously reported members, MERS (middle east respiratory syndrome) and SARS (severe acute respiratory syndrome) human coronaviruses. SARS-CoV-2 has caused a global pandemic since the first outbreak at the end of 2019. Therefore, targeting HSPA5 might benefit fighting against those diseases (7–11).

**Figure 1:**
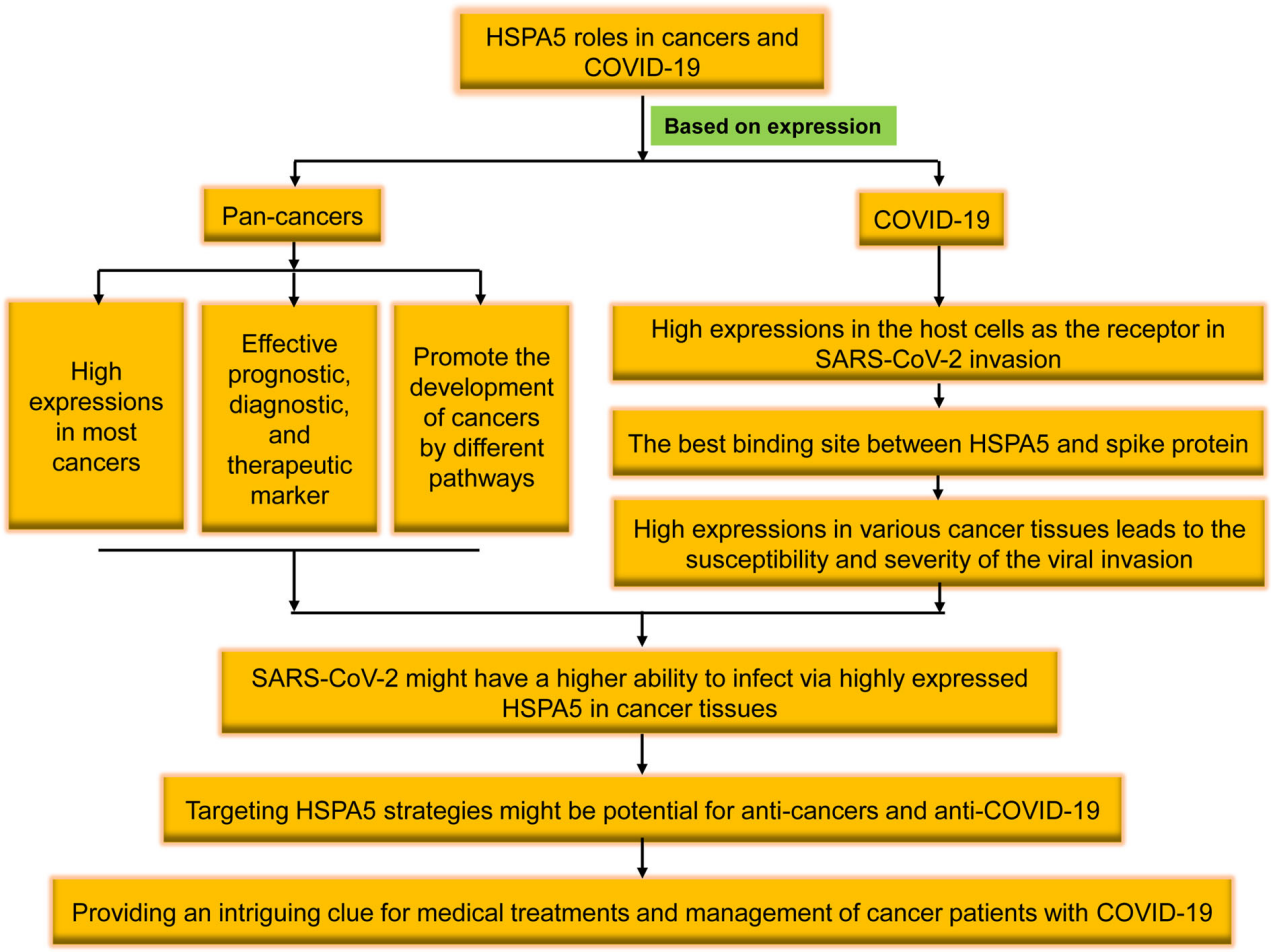
The brief flowchart of heat shock protein A5 (HSPA5) roles in pan-cancers and coronavirus disease 2019 (COVID-19). High expressed in the malignant tumors, HSPA5 likely plays a critical role in severe acute respiratory syndrome coronavirus 2 (SARS-CoV-2) invasion/attack in cancer patients, and targeting HSPA5 may not only implies the significance in clinical for both anti-COVID-19 and anti-cancers but also provides an intriguing clue for medical treatments and management of cancer patients with COVID-19 in the future.

In patients with prostate or ovarian cancer, the extracellular expositions of HSPA5 lead to product autoantibodies, which possess the ability of HSPA5 targeting. Since cell surface HSPA5 expression is associated with cancer and COVID-19, antibody strategies represent exciting target therapeutics (12). In this study, we will review the recent research progresses of cell surface HSPA5 functions, expressions, and the mechanisms/pathways of cancers and SARS-CoV-2 invasion. The therapeutic significance and prospects in cancers and SARS-CoV-2 entry by targeting HSPA5 will also be discussed (**Figure 1**).

## 2 HSPA5 in cancers

### 2.1 The *HSPA5* expression is significantly higher in most malignant cancers

**Figure 2:**
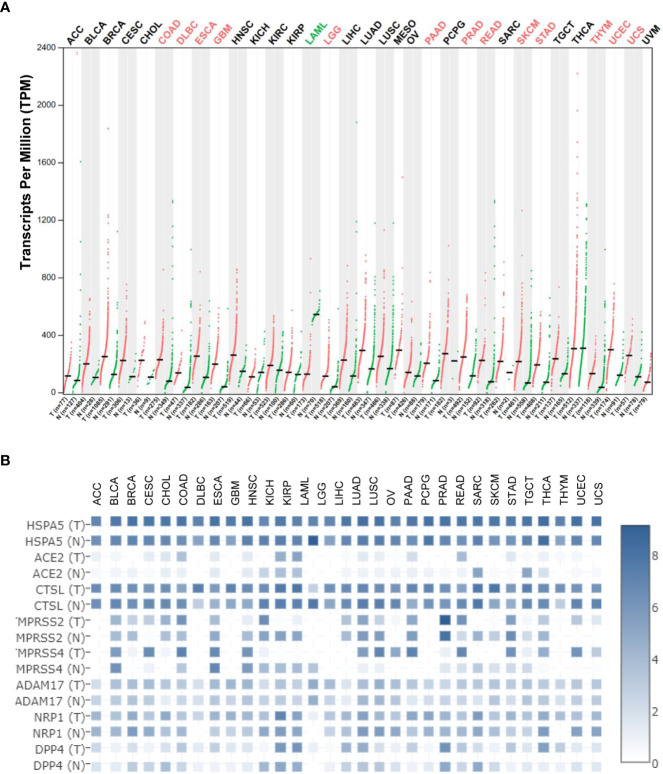
Heat shock protein A5 (HSPA5) expression comparisons in cancers and paired normal samples and its comparison among different entry proteins in pan-cancers. **(A)** HSPA5 expression in cancers and paired normal samples in 33 types of cancers. **(B)** Expression comparisons among HSPA5, angiotensin-converting enzyme 2 (ACE2), cathepsin L (CTSL), transmembrane protease serine 2 (TMPRSS2), transmembrane protease serine 4 (TMPRSS4), ADAM metallopeptidase domain 17 (ADAM17), neuropilin-1 (NRP1), and dipeptidyl peptidase-4 (DPP4) in both malignant cancers and corresponding normal samples in 31 types of cancers in The Cancer Genome Atlas (TCGA) datasets. The expression analysis was conducted in Gene Expression Profiling Interactive Analysis (GEPIA2) (http://gepia2.cancer-pku.cn/#analysis). “T” stands for tumor tissue, and “N” stands for the matched normal tissue.

Using the Gene Expression Profiling Interactive Analysis (GEPIA) and Human Protein Atlas (HPA) datasets, the *HSPA5* mRNA expression in different types of cancers and healthy tissues is high and the highest in thyroid carcinoma. Moreover, *HSPA5* expression was significantly upregulated in fourteen cancer types, including brain lower-grade glioma (LGG), cholangiocarcinoma (CHOL), colon adenocarcinoma (COAD), esophageal carcinoma (ESCA), lymphoid neoplasm diffuse large B-cell lymphoma (DLBC), glioblastoma multiforme (GBM), pancreatic adenocarcinoma (PAAD), rectum adenocarcinoma (READ), prostate adenocarcinoma (PRAD), stomach adenocarcinoma (STAD), thymoma (THYM), uterine corpus endometrial carcinoma (UCEC), skin cutaneous melanoma (SKCM), and uterine carcinosarcoma (UCS). In contrast, the expressions of *HSPA5* were remarkably downregulated only in acute myeloid leukemia (LAML) (**Figure 2A**) (13). These results implied that HSPA5 is an important marker for cancer which is highly expressed in the majority of malignant tumors and will be a helpful diagnostics and prognostic tool for cancer patients (14). Additionally, HSPA5 likely plays a critical role in SARS-CoV-2 invasion/attack in most cancer patients *via* tumor tissues if HSPA5 is highly expressed (13). HSPA5 expression showed a prognostic significance in patients with pancreatic ductal adenocarcinoma treated with neoadjuvant therapy versus those patients of surgery first (15). Recently, Wang et al. found that HSPA5 is upregulated in bladder cancer tissues and significantly associated with tumor progression and poor prognosis in the patients of bladder cancer (16).

### 2.2 The *HSPA5* expression is a prognostic, diagnostic, and therapeutic marker

By analyzing the database of The Cancer Genome Atlas (TCGA), the Tumor Immune Estimation Resource (TIMER) method, the Kaplan-Meier plotter, or Cox regression, Zhang et al. (17) concluded that HSPA5 is a marker for prognostics that correlates with immune infiltration of breast cancer. Dong et al. (18) revealed HSPA5 should be a marker for prognostics that correlates with immune infiltrates of another cancer type, thyroid carcinoma (THCA). CXCR4 belongs to the G protein-coupled receptor (GPCR) subfamily and is a cofactor facilitating the human immunodeficiency virus (HIV) entry into the CD4+ T cells. HSPA5 may be a target for an inducer of immunogenic cell death (19). Angeles-Floriano et al.(20) reported that HSPA5 and CXCR4, both expressed at the cell surface, are correlated with high-risk acute lymphoblastic leukemia for diagnostics in childhood. Tumor-associated antigens (TAAs) have been investigated as potential early diagnosis tools, Ma et al. (21) found that anti-p16 and anti-HSPA5 autoantibodies have the potential to be diagnostic markers for Hispanic hepatocellular carcinoma (HCC) patients. HSPA5 was reported to facilitate M2 macrophage polarization and lung tumor progression *in vitro* and *in vivo* (22). In addition, HSPA5 was reported to promote the response of osteogenesis and angiogenesis in periodontal ligament stem cells, thus considered a therapeutic target for the repair of the diseased periodontium (23). Thus HSPA5 might be a prognostic, diagnostic, and therapeutic marker (24).

### 2.3 HSPA5 promotes cancer cell viability, proliferation, and migration in cancers through different mechanisms/pathways

HSPA5 promotes cancer cell viability, proliferation, and migration in different tumor types. Mechanistically, Ha et al. (25) reported that targeting HSPA5 inhibits the expression of oncogenic KRAS protein and reduces the viability of cancer cells when they beard different KRAS variants. In comparison, Ning et al. (26) revealed that activation of HSPA5 ATPase suppresses migration by promoting ITGB4 degradation in A549 lung cancer cells. HDAC6 is a deacetylase that regulates cancer progression by modification of various substrates. By HDAC6 inhibition, the translocation of HSPA5 to the cell surface was blocked, thereby suppressing cell proliferation of cholangiocarcinoma (27). E3 ubiquitin ligase seven in absentia homolog 2 (Siah2) involves reactive oxygen species (ROS) generation under the conditions of hypoxia and hypoglycemia. Dixit et al. (28) reported that siah2-HSPA5 interaction modulates ROS and promotes cell proliferation of Helicobacter pylori-infected gastric epithelial cancers. HSPA5 was reported to determine the sensitivity of glioblastoma to UBA1 inhibition-induced UPR signaling and the death of cancer cells (29). HSPA5/Yorkie interactions promoted Ire1/Xbp1s pathway activation and aggravated epithelial-mesenchymal transition (EMT), migration, and invasion in triple-negative breast cancer (TNBC) (30).

Signaling pathways in CDK7/HSPA5 could contribute to tumor growth, invasion, and metastasis of osteosarcoma (31). Activating HSPA5/PERK signaling together with ER stress mediator TMTC3 promoted squamous cell carcinoma progression (32). DnaJ Heat Shock Protein Family (Hsp40) Member B11 (DNAJB11), a co-chaperone of HSPA5/BiP/GRP78 (33), was reported to promote the development of pancreatic cancer cells *in vitro* and *in vivo* by upregulating the HSPA5 expression and activating EGFR/MAPK pathway (34). Galectin−1, a small protein family member with an affinity for β-galactosides (35), was reported to bind HSPA5 to promote the proliferation and metastasis of gastric cancers *in vitro* and *in vivo* (36). Leucine zipper EF-hand-containing transmembrane protein-1 (LETM1) was disclosed to have its genomic deletions in Wolf–Hirschhorn syndrome (WHS) and revealed to regulate ion homeostasis, cell viability, mitochondrial morphology, as well as overexpression in different human cancers. LETM1/HSPA5 axis or HSPA5-LETM1 interaction was revealed to play roles in lung cancer progression (37). X-linked inhibitor of apoptosis-associated factor-1 (XAF1), a suppressor for stress-inducible tumors, was reported to drive apoptotic switches of ER stress response *via* destabilization of HSPA5 and ubiquitin E3 ligase CHIP (38). By stabilizing HSPA5, deubiquitinase USP11 was said to promote the chemoresistance of ovarian cancer (39). In cervix cancer, inhibiting the degradation of HSPA5 from activating FAK and overexpressing eukaryotic translation initiation factor 3D (EIF3D) were reported to increase stem cell-like properties and promote metastasis (40).

Homeobox (HOX) transcript antisense RNA (Hotair) is a long noncoding RNA significantly elevated in many cancers. Hotair/miRNA-30a/HSPA5/PD-L1 axis was reported to promote the progressions and immune escapes of laryngeal squamous cell carcinoma (LUSC) (41). By mediating the HSPA5-mediated autophagy and AKT/mTOR axis, radiosensitizer exosomal miR-197-3p was reported to inhibit nasopharyngeal carcinoma (NPC) progression and radioresistance (42). Salidroside, an extract from Rhodiola roots (molecular formula: C_14_H_20_O_7_), was reported to suppress the activation of NPC cells by targeting the axis of miR-4262/HSPA5 (43). In addition, down-regulating HSPA5 was reported to reverse pirarubicin resistance in TNBC through the pathway of p-AKT/mTOR and the mimics of miR-495-3p (44).

Ferroptosis was a new, non-apoptotic form of cell death recognized by iron-dependent lipid peroxidation. Both ferroptosis and unfolded protein response are critical factors in developing colorectal cancer (CRC). HSPA5 was reported to repress ferroptosis, thereby promoting CRC development by maintaining the stability of the GPX4 protein (45).

## 3 HSPA5 in SARS-CoV-2 invasion

### 3.1 SARS-CoV-2 entry proteins/receptors

Currently, it is well known that the SARS-CoV-2 virus enters the host cell mediated by the spike protein (S-protein) and host receptor(s) (46–48). Various entry related-proteins or host cell receptors/coreceptors have been identified for SARS-CoV-2 invasions. These include angiotensin-converting enzyme 2 (ACE2), Furin, BSG/CD147, cathepsin L (CTSL), transmembrane protease serine 2/4 (TMPRSS2/4), ADAM metallopeptidase domain 17 (ADAM17), neuropilin-1 (NRP1), dipeptidyl peptidase-4 (DPP4), and HSPA5 (49–58). HSPA5 shows high expression among above entry proteins even though CTSL expression shows comparable high in both malignant cancers and corresponding normal samples (**Figure 2B**), demonstrating the significance of viral invasion by HSPA5. Additionally, it was reported that HSPA5 is upregulated during SARS-CoV-2 infection while it acts as a pro-viral protein (59).

### 3.2 SARS-CoV-2 invasion through HSPA5

**Figure 3:**
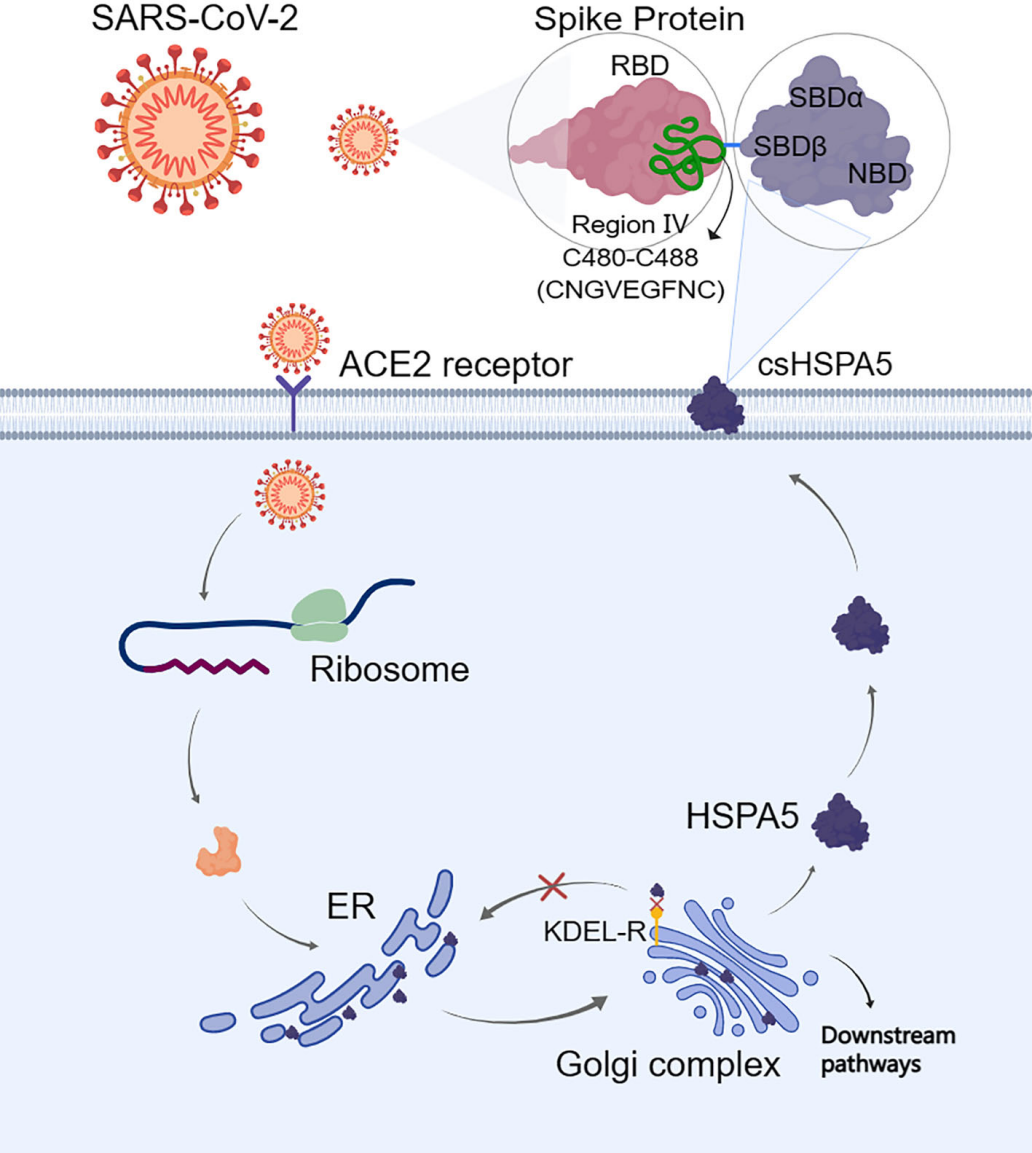
When the endoplasmic reticulum (ER) is stressed, heat shock protein A5 (HSPA5) is translocated to the cell surface (csHSPA5) and subsequently bound to the region IV (C480-C488) through the position of amino acids CNGVEGFNC in the spike protein (S-protein) of SARS-CoV-2, thus acted as a receptor/entry protein for virus entry. For the cell surface protein HSPA5, its SBDβ domain was predicted to bind to the spike protein. The image was created with MedPeer (www.medpeer.cn). ER, the endoplasmic reticulum; KDEL-R, the endoplasmic reticulum protein retention receptor; RBD, receptor binding domain; SBD, substrate binding domain.

The S-protein binding site to cell surface protein HSPA5 was first predicted by Ibrahim et al. (51) using molecular docking and structural bioinformatic strategies. They found that the binding sites are favorable at the regions of domain III (C391-C525) and IV (C480-C488) in the receptor-binding domain (RBD) of the S1 C-terminal domain. Also, they conclude that region IV (C480-C488) would be the main driving force site for the binding of cell surface HSPA5 protein. The best fit for the binding site to HSPA5 was the cyclic nine amino acid residues (CNGVEGFNC) of region IV in RBD, and the sequence of this amino acid was cyclic and surface-accessible and protrudes to the outside of the spike (60) (**Figure 3**). Equal average binding affinities of HSPA5 against the wildtype RBD and delta variants of the S-protein for SARS-CoV-2 were also revealed (61). The new variant spikes of 501.V2 and omicron are predicted to be tightly bound to HSPA5 more than the wildtype RBD (62, 63). While the ACE2 required TMPRSS2/4 to cleave the S-protein, the N-terminus nucleotide-binding domain (NBD) of the HSPA5 provided the energy for the SARS-CoV-2 entry (64). ACE2 requires HSPA5 for its translocation to the cell surface, and when HSPA5-depleted cells are tested, their ACE2 fails to be expressed on the cell surface but activates UPR markers(65).

Shinn et al. (66) reported the S-protein from SARS-CoV-2 can physically interact with cell surface HSPA5 protein in adipose tissue of COVID-19 patients with older age, obesity, and diabetes. Furthermore, Palmeira et al. (67) revealed that HSPA5 inhibitors interfered with SARS-CoV-2 entry through a virtual screening study. Therefore, HSPA5 could be an entry protein or coreceptor for the SARS-CoV-2 attachment and invasion (10, 51, 68, 69). Besides, the HSPA5 expression level was uncovered to be higher in the group of SARS-COV-2-positive patients compared with other groups (70).

### 3.3 The HSPA5 expression, susceptibility to infection, and severity of COVID-19

HSPA5 expression in various tissues may be tightly close to the susceptibility and severity of the viral invasion. Therefore, understanding the expression of HSPA5 in various malignant cancers and corresponding normal tissues is essential. In addition, organ dysfunctions, for example, acute respiratory distress syndrome (ARDS), acute cardiac injury, acute kidney injury (AKI), shock, and death, occur in severe events of COVID-19 (71, 72). Older people with comorbidities, for example, cardiovascular disease, diabetes, cerebrovascular disease, and high blood pressure, were also reported to suffer from severe COVID-19 (73, 74). Moreover, HSPA5 was reported to upregulate during SARS-CoV-2 infection in patient tissues and serum as a pro-viral protein (59).

The incidences of malignant tumors are increasing and are the general comorbidity with COVID-19 (75–77). Dysregulating the expression of HSPA5 in cancer tissues, particularly in the lungs, could influence the susceptibility to virus infection and its severity (13). A targeting strategy for HSPA5 might help develop and design new therapeutics against viral invasion associated with carcinomas during ER stress (69, 78, 79).

## 4 Associations between SARS-CoV-2 invasion and HSPA5 expression in cancers

Understanding the HSPA5 expressions, as mentioned above, and localizations of entry proteins/receptors of SARS-CoV-2 in host tissues can give insights into COVID-19 therapeutics for reducing the spread of COVID-19, viral replication, disease pathology, and disease severity. In addition to ACE2, other entry proteins like HSPA5 could act as receptors/coreceptors for SARS-CoV-2 entry (51, 80). The *HSPA5* mRNA levels were significantly higher than those of *ACE2* in both cancers and healthy individuals in most cancer types (**Figure 2B**). Moreover, in the normal lungs, the mRNA level for *HSPA5* was a 54.4-fold increase than that of ACE2, and in lung cancer, it was a 253-fold increase, implying that HSPA5 plays a vital role in SARS-CoV-2 invasion in cancer progression by the lungs (13).

Understanding the expression for HSPA5 is essential. Once again, HSPA5 expression showed high in almost healthy tissues and upregulated in most cancer tissues, suggesting that all the organs can be invaded, high susceptibility to SARS-CoV-2, and severity to diseases in those people bearing cancers (**Figure 2A**). Besides, high-expressed HSPA5 largely downregulated the overall survival of 7 types of cancer patients, such as adrenocortical carcinoma (ACC), bladder urothelial carcinoma (BLCA), head and neck squamous cell carcinoma (HNSC), kidney renal papillary cell carcinoma (KIRP), GBM, liver hepatocellular carcinoma (LIHC), and uveal melanoma (UVM).

Patients bearing malignant tumors are usually too weak and prone to more severe SARS-CoV-2 infection. When studying the expression level of ACE2, Lee et al. reported that patients of men with lung tumors likely have a high-risk COVID-19 condition (81). HSPA5 is expressed in male reproductive tissues that may facilitate the virus entry into the male reproductive tract, linking the SARS-CoV-2 and the HSPA5 could become a target of therapeutics to mitigate its harmful effects on male fertility (82). Our systematic review and meta-analysis showed that 7.15% of COVID-19 patients presented malignant tumor coincidental situation, and the rate of more severe events of patients with both COVID-19 and tumors was higher than that of all patients with COVID-19 (33.33% versus 16.09%, respectively, *p*<0.01) (13). Other systematic reviews and meta-analyses could also support coincidental cancer situations (83–85). Therefore, these data suggested that SARS-CoV-2 might have a high ability to infect highly expressed HSPA5 tissues, including cancer tissues. Altogether, HSPA5 expression implied the association, roles, and clinical significance in SARS-CoV-2 invasion in cancer patients.

## 5 HSPA5 targeting and the natural products in anti-cancer therapeutics and anti-SARS-CoV-2: the perspectives

Targeting HSPA5 strategies might be potential for anti-cancer therapeutics and anti-SARS-CoV-2 (7–11, 86). Presenting on the surface of cancer cells and not healthy cells *in vivo*, cell surface HSPA5 is an exciting target for antibody therapeutics, thereby providing valuable insights into the clinical values of HSPA5 antibodies for the prognosis and therapy of cancer and as anti-SARS-CoV-2 (12).

A combination of HSPA5-targeted and doxorubicin-loaded nanodroplets together with ultrasound was reported to be novel, potential theranostics in castration-resistant prostate cancer (87). Targeting HSPA5 was reported to sensitize reactive oxygen species (ROS) osteosarcoma cells to the therapy of pyropheophorbide-α methyl ester-mediated photodynamics (88). Anti-breast cancer drugs were predicted to bind to cs-HSPA5 in stressed cells. The cyclin-based kinases 4/6, abemaciclib, and ribociclib, and the effective anticancer agent, tunicamycin, maintained their binding affinity during 100 ns molecular dynamics simulation against the nucleotide-binding domain of cell-surface HSPA5 (89).

Host cell stress response may predict the infectivity of SARS-CoV-2 and the progression of COVID-19 disease (90). A decrease in HSPA5 expression may potentially prevent COVID-19, especially in cancer patients. Therefore, HSPA5 was implied as an anticancer drug target (11, 91, 92). On the cell surface, HSPA5 was earlier found to execute as an entry protein/coreceptor for virus internalization to associate with class I molecules of major histocompatibility complex (MHC). Thus antibodies can direct against both the N and C-terminus of HSPA5, majorly affecting the binding of the SARS-CoV-2 to the cell surface and its infectivity to liver cancer (93, 94). The virus entry for Borna disease was regulated by the association of HSPA5 with the cleaved N-terminus envelope glycoprotein GP1 (95). The antibody against the N-terminus of HSPA5 (N20) can interrupt GP1 binding to HSPA5 and reduce virus infection. Therefore, we could consider the potential of using HSPA5 inhibitors/antibodies for COVID-19 treatment (96). Indeed Shin et al. showed that the above-known inhibitors of HSPA5 interfered with the SARS-CoV-2 infection through virtual screening studies (66). Two of these drugs, Ponatinib and Bosutinib, are SRC inhibitors and are patented as capable of blocking the expression of HSPA5.

Natural products have been shown to disrupt the attachment of SARS-CoV-2 to stressed cells, which is also worth further investigating (69, 97). These products can interfere with SARS-CoV-2 attachment through the substrate-binding domain β (SBDβ) of HSPA5 on the host-cell membranes (69). For example, in addition to anti-cancerous effects, terpenoids of the Chaga mushroom (*Inonotus obliquus*), mainly for Oleanolic acid and Inonotsulide A, had high affinities toward the HSPA5 SBDβ (98). Previous *in silico* studies also revealed that terpenoids of the Chaga mushroom might interfere with SARS-CoV-2 recognition by the host cells by binding the viral spike protein (99). Based on the values of binding affinities, phytoestrogens (Biochanin A, Diadiazin, Genistein, and Formontein) and estrogens are the best to bind HSPA5, thus could disrupt SARS-CoV-2 attachments. We thus could use these small molecules from mushrooms or herbs as anti-COVID-19 drugs for people with higher risks of cell stress, like cancer patients, elders, and front-line medical staff. Therefore, targeting HSPA5 expression by natural products may imply the significance in clinical for both anti-COVID-19 and anti-cancers in the future.

Moreover, SARS-CoV-2 was frequently mutated. Interestingly, some mutated variants of the S-protein RBD in SARS-CoV-2 are predicted to be tightly bound to cell surface HSPA5 more than that of the wildtype S-protein RBD (62, 63, 100). The binding affinities of both ACE2 and HSPA5 against the SARS-CoV-2 spikes of the wildtype and the alpha, beta or gamma, delta, delta+, C36, lambda, and omicron variants were predicted by Elfiky et al. (101). Both lambda and omicron variant spikes showed enhanced average binding affinities against HSPA5. This might be a key for the design of inhibitors to interfere with SARS-CoV-2 attachments and entry to the host cell by disrupting S-protein/HSPA5 binding (62, 63).

Altogether, we reviewed the high expression of HSPA5 in the most cancers and the possibility of being invaded by the virus as a new coronavirus receptor. Targeting HSPA5 may not only implies the significance in clinical for both anti-COVID-19 and anti-cancers but also provides an intriguing clue for medical treatments and management of cancer patients with COVID-19 in the future (**Figure 1**).

## Author contributions

JuF conceived and supervised the study. JiF, TL, and JuF collected literature, analyzed, and interpreted data. JuF wrote the draft manuscript. JiF, TL, AE, and JF reviewed and approved the final manuscript. All authors contributed to the article and approved the submitted version.
